# Interventional Strategies for Alleviating Severe Abdominal Pain in Chronic Pancreatitis and Abdominal Cancer: A Case Report on the Use of Splanchnic Nerve Radiofrequency Ablation and Erector Spinae Plane Block

**DOI:** 10.7759/cureus.63726

**Published:** 2024-07-03

**Authors:** Dnyanshree Wanjari, Amreesh Paul, Nikhil Bhalerao, Urvi Sawant

**Affiliations:** 1 Department of Anaesthesiology, Jawaharlal Nehru Medical College, Datta Meghe Institute of Higher Education and Research, Wardha, IND

**Keywords:** radiofrequency ablation, splanchnic nerve block, pancreatic cancer, chronic pain management, erector spinae plane block (espb), cancer-related pain

## Abstract

A cancer diagnosis marks the beginning of a difficult path filled with a profound battle against the excruciating pain associated with the illness. Cancer-related pain, which is complex and emotionally distressing, presents unique challenges in terms of treatment. Abdominal cancers and metastases frequently result in severe and unmanageable pain that does not respond well to traditional medications. In such situations, interventions like neurolysis and radiofrequency ablation of the splanchnic nerves and celiac plexus have emerged as effective strategies, providing enhanced pain relief and reducing the need for narcotic painkillers.

In this case report, we describe a case of a 38-year-old man with a longstanding history of chronic pancreatitis with a polypoid growth close to the ampulla in the duodenal bulb. The patient was given pain medications to alleviate the pain, but the severe stomach pain, vomiting, and fever persisted. Imaging tests supported the diagnosis and showed chronic pancreatitis, a continuing inflammatory process, and a periampullary adenocarcinoma. The patient had significant pain while being positioned prone for the diagnostic block, hence an erector spinae plane block was done before the radiofrequency ablation. The patient received radiofrequency ablation at the T11 and T12 levels after receiving a diagnostic splanchnic nerve block, significantly reducing pain. The effectiveness of these interventional procedures in enhancing the patient's quality of life and decreasing their dependence on narcotic drugs was highlighted by follow-up visits at two, four, and six months that revealed little to no discomfort. This instance emphasizes the importance of considering neurolysis and radiofrequency ablation as essential alternatives for treating severe abdominal pain brought on by chronic pancreatitis and abdominal cancer.

## Introduction

Being diagnosed with cancer is an upsetting moment that ushers people into a difficult road. The first challenge is surviving cancer, but it frequently requires a significant fight against the awful agony that comes along with the illness, intensifying the total suffering. As the cancer spreads, this pain is dynamic, fluctuating in severity, and frequently accompanied by acute bouts. The International Association for the Study of Pain defines pain as an unpleasant, sensory, complex, and emotional experience linked to actual or potential tissue damage [1]. Pain is recognized by the Joint Commission on Accreditation of Healthcare Organizations as the fifth vital sign [2]. Pain is one of the most common symptoms associated with cancer. Cancer-related pain differs from other types of discomfort in its unique way [3]. A widely recognized regimen for controlling pain has been devised by the World Health Organization (WHO), starting with non-steroidal anti-inflammatory medicines and moving on to weak opioids and potent opioids when needed [4]. However, metastases or cancers of the abdomen can cause excruciating pain resistant to treatment with traditional pharmaceuticals. Additionally, many medications may have unwanted side effects that patients encounter. Even though narcotic and non-narcotic medication combinations are routinely used, the results sometimes fall short of expectations. Interventional procedures are necessary when pharmacological therapies fail. Improved quality of life, pain alleviation, and a decreased need for drugs are all benefits of radiofrequency ablation (RFA) of the celiac plexus and splanchnic nerves, as well as neurolysis. The celiac plexus plays a vital role in transmitting painful impulses from the upper abdominal viscera, which is situated deep within the retroperitoneum and surrounds the anterolateral surface of the aorta [5]. Nearly a century ago, percutaneous injection methods to block the celiac plexus and splanchnic nerves were developed, and neurolysis gained popularity for treating gastrointestinal cancer pain in the middle of the 20th century. The tumor may have altered the anatomy of the celiac plexus, and there may also have been enlarged lymph nodes, making these obstructions challenging to remove. Consequently, effective alternatives for providing these patients with greater pain relief include neurolysis and RFA of the splanchnic nerves [6]. The erector spinae plane block also offers good temporary pain relief for patients suffering from chronic pancreatitis, and having difficulty in providing position for diagnostic blocks and RFA [7].

## Case presentation

A 38-year-old male complained of pain in his abdomen for four months, gradual in onset and progressive in nature, associated with episodes of vomiting and fever for one month. The patient had no bladder or bowel complaints, abdominal trauma, cough, or breathlessness. The patient has been a known alcoholic for the past 10 years, with a history of tobacco consumption. The patient had no known co-morbidity. An ultrasonography of the abdomen was done that revealed a heterogeneously appearing pancreas, with the pancreatic duct mildly dilated, measuring 4.4 mm in diameter, suggestive of chronic pancreatitis. The patient was evaluated and was found to have an amylase level of 204.5 IU/L and a lipase level of 864.3 IU/L. Contrast-enhanced computed tomography (CECT) of the abdomen revealed features suggestive of chronic pancreatitis with an acute inflammatory process seen in the uncinate process and a few pseudocysts around the pylorus and D1 segment of the duodenum. Magnetic resonance cholangiopancreatography (MRCP) was done, and it revealed chronic pancreatitis with focal stricture in the proximal common bile duct. Upper gastrointestinal endoscopy revealed an enormous peri-ampullary polypoidal friable growth in the duodenal bulb, almost occluding the duodenum.

**Figure 1:**
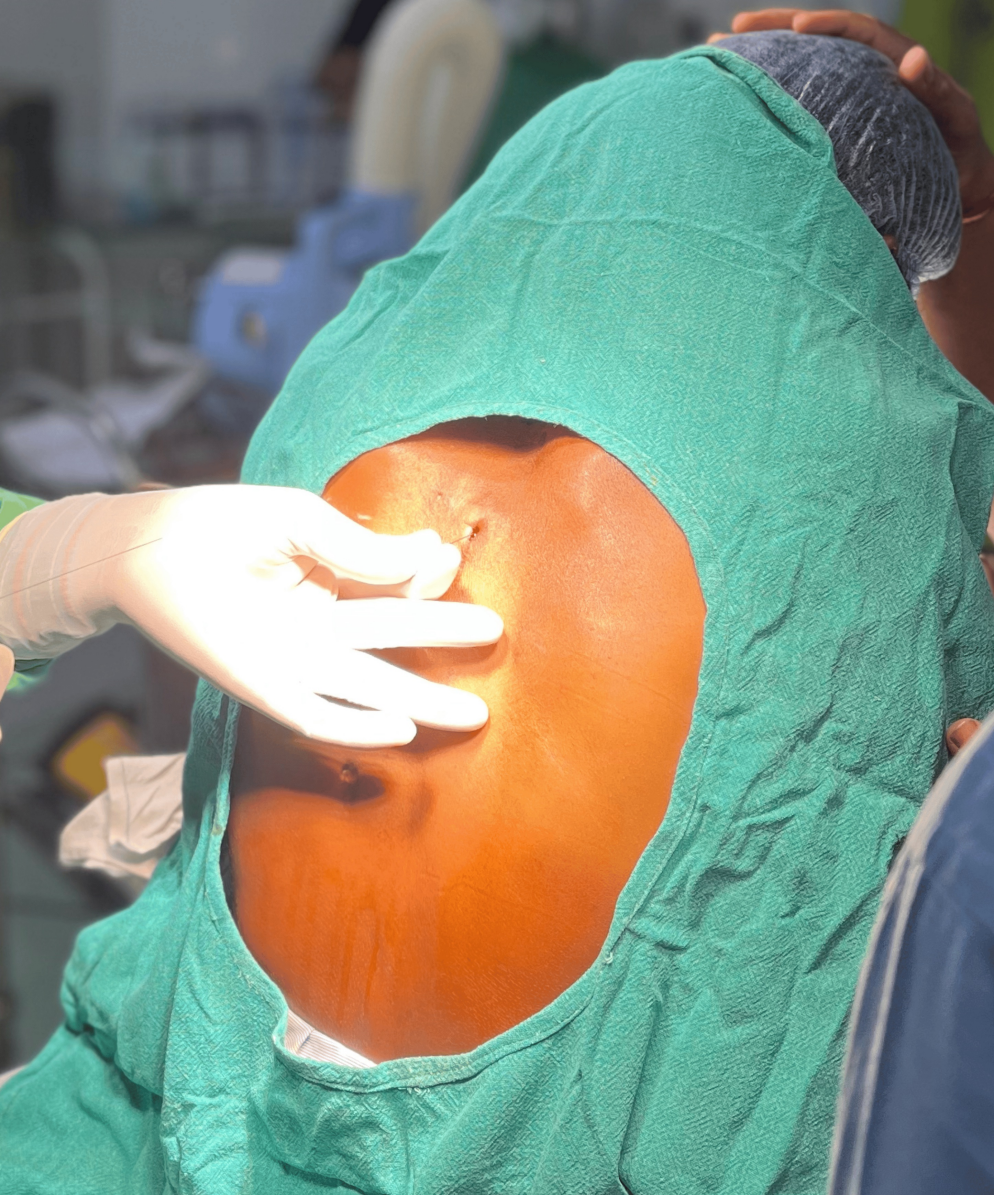
Administration of erector spinae plane block.

A specimen biopsy showed chronic lymphohistiocytic infiltrate with prominent eosinophilia, with marked atypical cells, and was positive for adenocarcinoma. Hence, the patient was diagnosed with peri-ampullary adenocarcinoma. The patient was referred to the department of anesthesiology and pain management because of chronic pain. The patient was examined and was started on tapentadol 100 mg twice a day, amitriptyline 10 mg at bedtime, and paracetamol 1 g three times a day. The patient was counseled for splanchnic nerve diagnostic block and RFA. Laboratory investigations revealed a normal complete blood count and coagulation profile. On the day of the procedure, the patient was kept nil per os for six hours, shifted to the cath lab, monitors attached, and vitals noted. Under strict aseptic precautions, with the patient in the prone position, local anesthesia was injected, and a splanchnic nerve block was done with lignocaine 1% bilaterally at the T11 and T12 levels after adequate contrast spread under fluoroscopic guidance. The patient had terrible pain before block administration due to the positioning, but the patient experienced near-complete pain relief immediately after the procedure. After the procedure, the patient was shifted to the postoperative room, and vitals were monitored. After two days, RFA of the splanchnic nerve was planned. As the time required for performing RFA is considerably more, and due to the patient having pain during prone positioning, it was decided to administer a landmark-guided erector spinae block to the patient. Under strict aseptic precautions, with the patient sitting, the spinous process of the right T8 vertebrae was identified using the landmark-guided method, and the skin at about 3 cm lateral from the spinous process was infiltrated with lignocaine 1%. A 23G spinal needle was inserted at this point and was advanced until the transverse process was hit at about 3.5 cm from the skin as seen in Figure 1. At this point, the needle was between the erector spinae muscle and the transverse process, and 15 ml of bupivacaine (0.25%) was inserted after negative aspiration of blood and cerebrospinal fluid. The same procedure was done on the left side too. The patient was then positioned prone, and he did not experience any pain.

**Figure 2:**
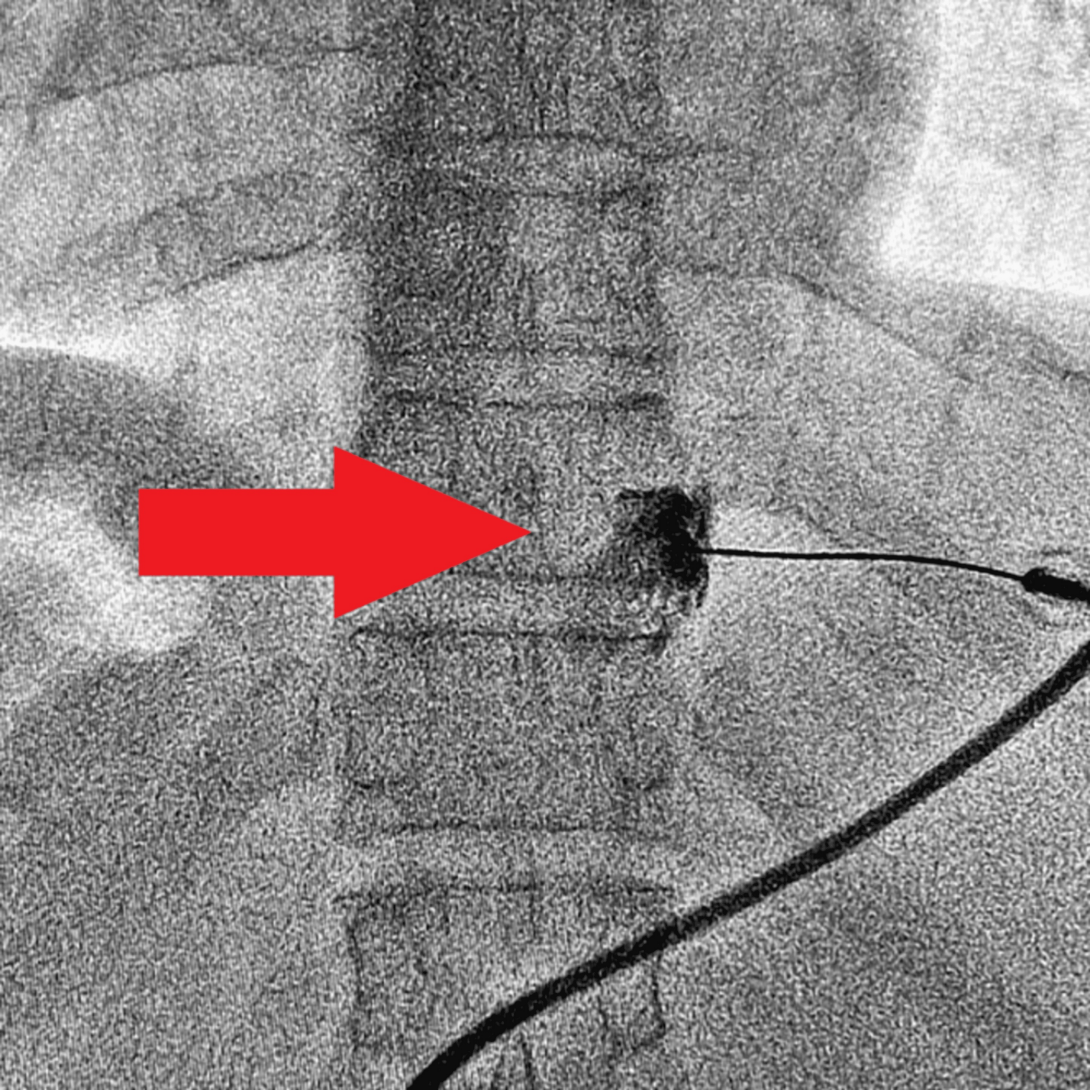
Fluoroscopic image showing contrast spread at T11 level on the right side.

**Figure 3:**
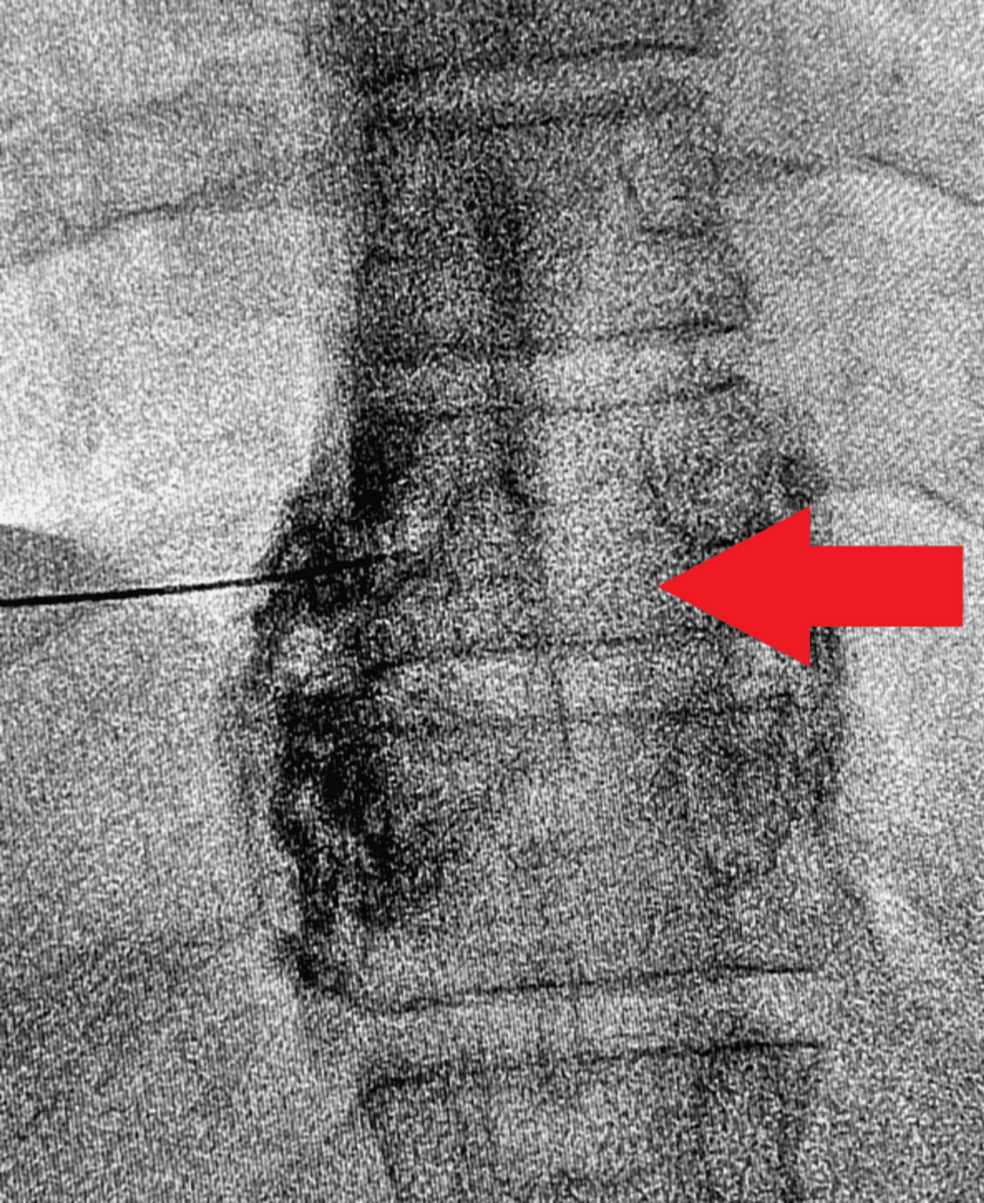
Fluoroscopic image showing contrast spread at T11 level on the left side.

After adequate pain relief, under fluoroscopic guidance, RFA of the splanchnic nerves was done at T11 and T12 levels bilaterally with a 15 cm Chiba needle with a 10 mm active tip cannula. Figures 2, 3 show the contrast spread achieved after needle placement under fluoroscopic guidance on both sides.

A sensory and motor test was used to determine which nerves were involved. The following settings were applied to the sensory stimulation: 50 Hz frequency, 1 ms pulse width, and 1.0 V of voltage. It was anticipated that the patient would have pressure, pain, or overall discomfort in the lower abdomen, and occasionally in the lower back. In case this did not happen, the needle was moved a few millimeters forward or backward until the right sensory reaction was experienced. The parameters for motor stimulation were voltage up to 2.5 V, pulse width of 1 ms, and frequency of 2 Hz. Throughout the test, there should be no intercostal muscular contractions. The electrode was advanced a few millimeters anteriorly if contractions were felt, which would have indicated that the electrode's active tip was too near to the intercostal nerve. Two monopolar radiofrequency lesions were made concurrently at 80°C during the lesion procedure. The lesions had a total duration of 90 seconds, including the ramp time, and a ramp period of 15 seconds to reach the maximum temperature. To lessen postoperative tissue edema and discomfort, 2.5 mL of a 10 mL solution containing 2 mL of dexamethasone (4 mg/mL) and 8 mL of ropivacaine (7.5 mg/mL) was given as soon as each lesion was created. The procedure was uneventful, and the patient was shifted to the postoperative room for monitoring. The patient was discharged on post-procedure day eight. The patient had almost no pain on the day of discharge. The patient was followed up on months two, four, and six and was found to have zero to negligent pain during these visits.

## Discussion

Interventional methods, including splanchnic nerve blocks and RFA, can be used to provide comfort and pain relief to patients suffering from chronic pain due to pancreatic cancer. They promise a better quality of life, efficient pain alleviation, and a decreased need for medicine. The discussion emphasizes the celiac plexus' anatomical significance as a critical node for communicating discomfort signals from the upper abdominal viscera. It is positioned deep within the retroperitoneum [8]. The patient had significant pain during positioning for the diagnostic block, and hence a landmark-guided bilateral erector spinae plane block (ESPB) was administered to alleviate pain during positioning for the RFA [9]. Decreasing the need for opioids, and providing long-lasting pain relief provides much-needed comfort to such patients [10].

In this case study, we address the effectiveness and safety of ESPB and splanchnic nerve RFA in treating patients with severe stomach discomfort who also had chronic pancreatitis and abdominal cancer. The use of ESPB to treat abdominal pain in cases of chronic pancreatitis and abdominal cancer is supported by recent research. Gopinath et al. reported a case in which uncontrollably severe abdominal pain associated with pancreatitis was successfully managed by a single bolus of local anesthetic in the erector spinae muscle plane [11]. Similar to this, Mantuani et al. showed that ESPBs in the emergency room were effective in relieving acute pancreatitis pain [12].

Additionally, neurolytic ESPB has been investigated, especially for excruciating cancer pain. Neurolytic ESPBs with 5%-8% phenol have been shown in multiple case reports to provide effective pain relief for periods ranging from two weeks to five months [13-15]. However, this procedure should only be used with caution due to the possibility of major side effects, including paraplegia, autonomic dysfunction, and bowel-bladder dysfunction.

In the treatment of severe abdominal pain, ESPB and splanchnic nerve RFA may work in conjunction. While splanchnic nerve RFA can produce longer-lasting analgesia, ESPBs can offer substantial, instantaneous pain relief. Patients with chronic pancreatitis and stomach cancer, whose pain management is frequently intricate and multifaceted, may find this combined approach especially helpful.

## Conclusions

In conclusion, this case study emphasizes the mental and physical anguish that cancer patients experience as well as the difficulties in coping with the unbearable agony that comes with the disease. We used a combination of splanchnic nerve blocks and RFA for pain alleviation. The patient experienced minimal pain during RFA due to the administration of a bilateral erector spinae plane block. This example highlights the significance of a comprehensive approach to diagnosis and treatment in complicated situations with periampullary adenocarcinoma and chronic pancreatitis.
